# Timing of percutaneous coronary intervention and risk of new‐onset acute ischemic stroke in non‐ST elevation myocardial infarction: A retrospective cohort study insight into the National Inpatient Sample Database (2016–2019)

**DOI:** 10.1002/hsr2.70029

**Published:** 2024-09-18

**Authors:** Bo Shi, Xueping Ma, Congyan Ye, Rui Yan, Shizhe Fu, Kairu Wang, Mingzhi Cui, Ru Yan, Shaobin Jia, Guangzhi Cong

**Affiliations:** ^1^ Institute of Medical Sciences General Hospital of Ningxia Medical University Yinchuan China; ^2^ School of Clinical Medicine Ningxia Medical University Yinchuan China; ^3^ Institute of Cardiovascular Medicine General Hospital of Ningxia Medical University Yinchuan China; ^4^ Department of Cardiology, General Hospital of Ningxia Medical University Ningxia Medical University Yinchuan China

**Keywords:** new‐onset acute ischemic stroke, non‐ST elevation myocardial infarction, percutaneous coronary intervention, timing of percutaneous coronary intervention

## Abstract

**Background and Aims:**

For patients with high‐risk non‐ST elevation myocardial infarction (NSTEMI), current guidelines recommend an early invasive strategy within 24 h. New‐onset acute ischemic stroke (NAIS) is a rare but fatal complication of percutaneous coronary intervention (PCI). However, the effect of the timing of PCI and the risk of NAIS in NSTEMI is poorly defined.

**Methods:**

Patients with NSTEMI who underwent PCI were queried from the National Inpatient Sample Database (2016–2019) and stratified into three groups: early (<24 h), medium (24–72 h), and late (>72 h) PCI. Multivariate logistic regression models were used to determine the association between timing of PCI and NAIS.

**Results:**

Among 633,115 weighted hospitalizations, patients in the late PCI group had a higher incidence of NAIS (1.3%) than those in the early (0.67%) and medium (0.71%) PCI groups. Patients undergoing late PCI were older, more likely to be female, and had a greater incidence of comorbidities (e.g., diabetes mellitus, chronic pulmonary and renal illness, and atrial fibrillation) than those undergoing early or medium PCI. After adjustment, only late PCI was significantly associated with a 54% increased NAIS risk (adjusted odds ratio: 1.54 [95% confidence interval: 1.29–1.84]). Additionally, there was heterogeneity in the magnitude of risk by age and sex. Younger people (<65 years) (*p* for interaction <0.001) and men (interaction‐value *p* = 0.040) were more likely to encounter NAIS.

**Conclusion:**

Late PCI was associated with a higher risk of NAIS than early PCI, particularly among men and those aged <65 years.

## 1 INTRODUCTION

New‐onset acute ischemic stroke (NAIS) is a rare but often fatal complication of percutaneous coronary intervention (PCI).^1^, ^2^ The incidence of post‐PCI NAIS has increased significantly among patients undergoing PCI for non‐ST elevation myocardial infarction (NSTEMI) over the past decade (from 0.5% to 1.0%).^3^ NAIS is associated with major patient complications, protracted hospitalizations, expensive medical expenses, and poor long‐term results.^4^ Independent predictors of periprocedural stroke include older age, history of prior stroke, diabetes mellitus, renal diseases, atrial fibrillation, and cardiogenic shock.^1^, ^5^, ^6^ The HALP (hemoglobin, albumin, lymphocyte, and platelet) score is a novel predictor of in‐hospital mortality for patients with ST elevation myocardial infarction and adverse outcomes in acute ischemic stroke.^7^, ^8^ Although these factors cannot be prevented, the timing of PCI after a patient is admitted to the hospital is determined by a physician.

Despite agreement on the necessity for urgent primary PCI in patients with ST‐elevation myocardial infarction (STEMI), ambiguity remains regarding the optimal timing of coronary intervention following NSTEMI. Variations in the clinical presentation of NSTEMI complicate the decisions relating to the timing of intervention.^9^ Current guidelines on the management of NSTEMI categorize intervention as immediate (within 2 h), early (within 24 h), or as a selected invasive strategy, and patients with high‐risk factors are recommended to undergo intervention within 24 h.^10^

Early intervention may help to avoid the occurrence of ischemic events while the patient waits for a delayed procedure.^11^ A meta‐analysis including 17 randomized clinical trials (RCTs) showed that an early invasive strategy (3.43 h [1.47–5.40 h]) did not reduce NAIS risk compared with a delayed invasive strategy (41.3 h [29.3–53.2 h]) in all patients with NSTEMI. The RCTs included in the meta‐analysis used different time intervals for early and delayed invasive strategies, and in one‐third of the trials, the time intervals used for delayed invasive strategies were quite early (median <24 h). Therefore, the therapeutic benefits obtained from executing an invasive strategy within 24 h would reduce the increased risk of stroke associated with delayed invasive strategies.^12^, ^13^ A large retrospective cohort study including 11,852 consecutive patients who underwent PCI for NSTEMI divided the patients into three groups by time from symptom onset to PCI (<24 h; 24–72 h; and >72 h) and showed no significant difference in the incidence of NAIS. However, this study ignored confounding and bias factors, which reduced the reliability and validity of the results.^14^

Here, we hypothesized that late PCI is associated with increased NAIS risk in NSTEMI compared with early PCI. We aimed to evaluate the relationship between the timing of PCI and the incidence and risk of NAIS in patients with NSTEMI following admission in a nationally representative database.

## 2 METHODS

### 2.1 Data source

The Agency for Healthcare Research and Quality sponsors the National Inpatient Sample (NIS) database, the largest all‐payer database in the United States, as part of the Healthcare Cost and Use Project, representing a stratified sample of 20% of all community hospital discharges in the United States. Additional discharge weights for each patient's record may be used to obtain national weighted estimates. The yearly compilation of the NIS allows for the analysis of sickness trends over time using the data. From 2016 to 2019, patients were selected from the NIS provided that they had suitable International Classification of Diseases, 10th Revision, Clinical Modification (ICD‐10‐CM) diagnoses and Procedure Coding System (PCS). This retrospective cohort study was adhered to the Strengthening the Reporting of Observational Studies in Epidemiology reporting guidelines. Because of the public nature of the NIS database (www.hcup-us.ahrq.gov) and the lack of any individually identifiable information, the Institutional Review Board waived the requirement for informed consent under the Health Insurance Portability and Accountability Act.

### 2.2 Study population

**Figure 1:**
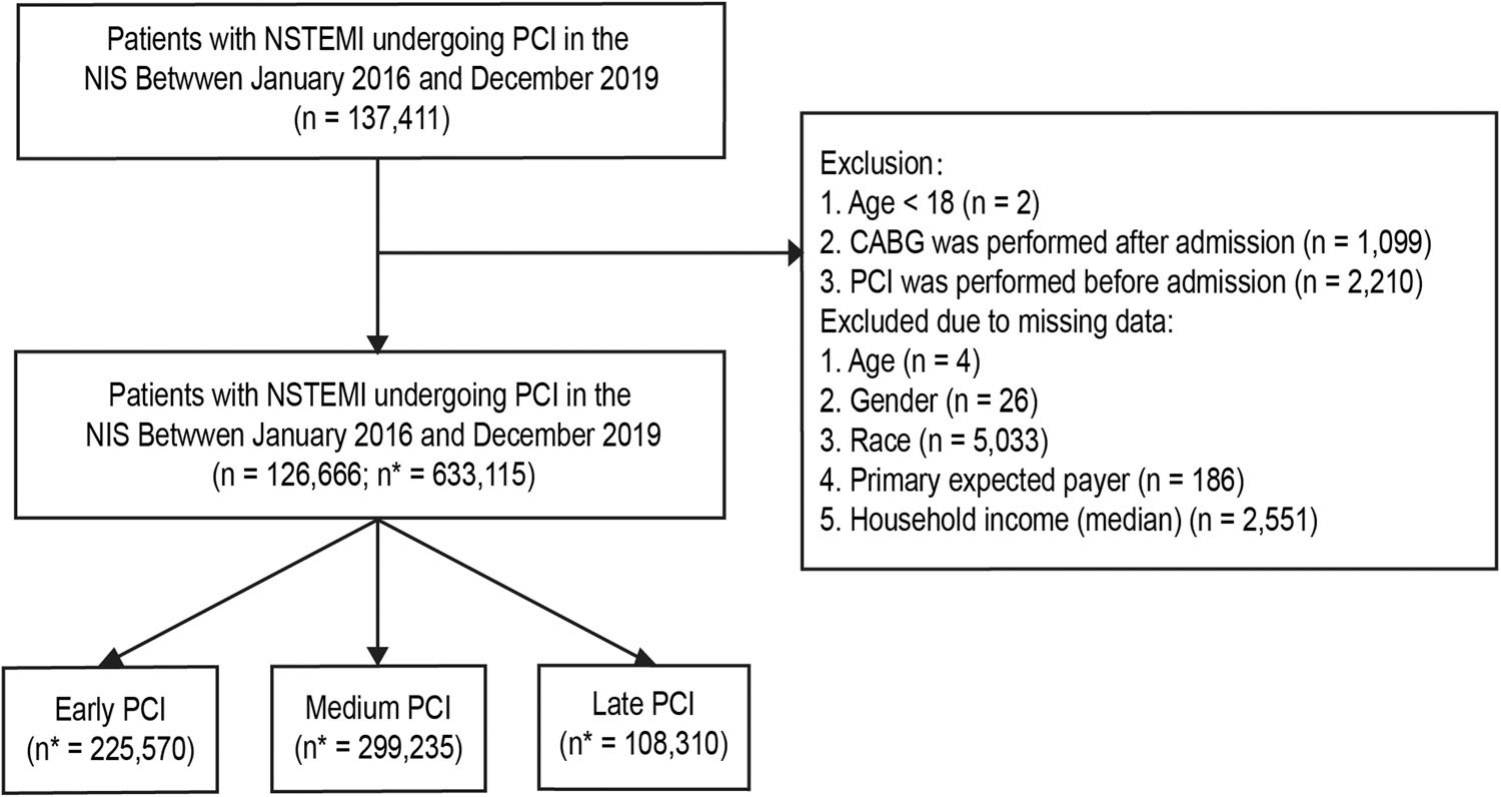
Flowchart of the study. CABG, Coronary artery bypass grafting; NIS, National Inpatient Sample; NSTEMI, non‐ST elevation myocardial infarction; PCI, Percutaneous coronary intervention. The symbol * indicates a weighted estimate.

From January 2016 to December 2019, a retrospective cohort analysis of admissions was performed using the NIS database to identify adult patients (>18 years old) with a primary diagnosis code of NSTEMI (ICD‐10‐CM code I214) who underwent PCI (ICD‐10‐PCS codes 0270X, 0271X, 0272X, and 0273X). The other ICD‐10‐CM diagnoses and procedure codes that were used to identify these patients are included in Table S1. Records that lacked crucial variables (age, sex, race, primary expected payer, and household income) or influenced the results (coronary artery bypass grafting performed after admission and PCI performed before admission) were excluded (Figure 1). The entire cohort of hospitalizations who met the inclusion criteria were classified into three groups based on when PCI was conducted after admission: early (<24 h), medium (24–72 h), and late PCI (>72 h).

### 2.3 Study outcomes

New‐onset acute ischemic stroke (NAIS) (ICD‐10‐CM code I63X) was the key outcome of interest. We also evaluated the hospital length of stay and total hospital charge. Temporal trends in NAIS incidence were also evaluated during the research period.

### 2.4 Statistical analysis

The NIS discharge weights were used to obtain weighted national estimates. For continuous variables, data is presented as survey‐weighted mean (95% confidence interval [CI]), and the p‐value was calculated using survey‐weighted linear regression. For categorical variables, data is reported as survey‐weighted percentages (95% CI), and the p‐value was calculated using the survey‐weighted Chi‐square test. The 95% CI was calculated by training a logistic regression model and constructing a Wald‐type interval on a log‐odds scale. Multiple logistic regression models were used to examine the connections between PCI timing and NAIS. Non‐adjusted and multivariate‐adjusted models were used, with variables adjusted for age, sex, race, weekday admission, primary expected payer, median household income, family history of coronary artery disease (CAD), prior stroke or transient ischemia attack (TIA), prior myocardial infarction (MI), prior PCI, prior CABG, smoking, dyslipidemia, obesity, diabetes mellitus, hypertension, carotid artery disease, congestive heart failure, peripheral vascular disease, chronic pulmonary disease, chronic renal disease, valvular disease, atrial fibrillation, cardiogenic shock, number of treated lesions, and use of intra‐aortic balloon pump (IABP). These covariates are factors known to affect NAIS and were chosen a priori.^1^, ^3^, ^5^, ^6^, ^15^ Stratification analyses were used to determine whether the time of PCI had a different impact on different subgroups, and their interactions were checked. The timing of PCI each year throughout the research period was also used to calculate the incidence of NAIS and compare trends in the three groups of patients over time. The statistical tools R (The R Foundation; http://www.r-project.org; version 4.2.0) and Empower (R) (www.empowerstats.com, X&Y solutions, Inc., Boston, MA) were used to analyze the data. P‐values were two‐sided, with a threshold of significance of 0.05.

## 3 RESULTS

### 3.1 Baseline characteristics

Between January 2016 and December 2019, 633,115 consecutive patients were included in the study, comprising 225,570 (35.60%) who underwent PCI within 24 h (early PCI), 299,235 (47.30%) who underwent PCI between 24 and 72 h (medium PCI), and 108,310 (17.10%) who underwent PCI > 72 h (late PCI). The patients’ baseline characteristics are shown in Table 1. The patients in the late PCI group were older, more likely to be female and black, and had a greater probability of having Medicare/Medicaid as their major payer (74.33% vs. 59.11% and 62.66%; *p* < 0.001) and belonging to the lowest quartile median family income (34.73% vs. 28.03% and 30.11%; *p* < 0.001). The patients in the late PCI group also had a greater prevalence of diabetes mellitus, congestive heart failure, chronic pulmonary and renal disease, atrial fibrillation, and cardiogenic shock, requiring more IABP support (all *p* < 0.001) (Table 1). Compared to the early and medium PCI groups, the late PCI group exhibited a significantly higher proportion of cases involving multi‐vessel treatment (two or more vessels) for coronary lesions (Table 1).

**Table 1:** Baseline demographics, comorbidities, and hospital characteristics of patients with non‐ST elevation myocardial infarction (NSTEMI) undergoing PCI among the early PCI, medium PCI, and late PCI groups.

	Total (%) 95% CI	Early PCI (%) 95% CI	Medium PCI (%) 95% CI	Late PCI (%) 95% CI	*p*‐value
n	633,115	225,570	299,235	108,310	
Patient demographics					
Age, mean, y (SD)	65.74 (65.61, 65.86)	64.24 (64.08, 64.40)	65.70 (65.55, 65.84)	68.98 (68.74, 69.23)	<0.001
Female	34.67 (34.27, 35.06)	32.40 (31.74, 33.06)	34.54 (33.76, 35.33)	39.75 (38.91, 40.59)	<0.001
Race					<0.001
White	77.13 (76.12, 78.11)	77.73 (76.85, 78.58)	77.35 (76.32, 78.34)	75.31 (73.23, 77.27)	
Black	10.24 (9.58, 10.94)	9.63 (9.16, 10.12)	10.19 (9.35, 11.08)	11.65 (10.67, 12.71)	
Hispanic	7.47 (7.10, 7.86)	7.33 (6.83, 7.87)	7.67 (7.11, 8.28)	7.19 (6.56, 7.88)	
Other	5.16 (4.74, 5.61)	5.31 (4.96, 5.69)	4.79 (4.31, 5.33)	5.85 (5.11, 6.68)	
Primary expected payer					<0.001
Medicare/Medicaid	63.39 (62.83, 63.95)	59.11 (58.51, 59.71)	62.66 (61.76, 63.56)	74.33 (73.42, 75.23)	
Private insurance	28.02 (27.39, 28.66)	32.13 (31.45, 32.82)	28.51 (27.73, 29.30)	18.11 (17.29, 18.97)	
Self‐pay	4.76 (4.30, 5.27)	5.26 (4.73, 5.86)	4.80 (4.37, 5.27)	3.60 (3.05, 4.23)	
No charge/other	3.82 (2.96, 4.93)	3.50 (3.05, 4.01)	4.02 (2.92, 5.51)	3.96 (2.84, 5.48)	
Weekend admission	25.17 (24.75, 25.60)	16.06 (15.14, 17.02)	32.20 (31.74, 32.66)	24.75 (23.75, 25.78)	<0.001
Household income (median)					<0.001
0–25th percentile	30.16 (28.86, 31.49)	28.03 (26.65, 29.45)	30.11 (28.64, 31.62)	34.73 (33.33, 36.15)	
26–50th percentile	27.58 (26.87, 28.31)	27.78 (26.89, 28.70)	27.36 (26.64, 28.08)	27.80 (26.76, 28.85)	
51–75th percentile	24.12 (23.19, 25.07)	24.85 (23.71, 26.03)	24.41 (23.28, 25.57)	21.78 (20.93, 22.66)	
76–100th percentile	18.14 (17.09, 19.24)	19.33 (18.20, 20.52)	18.12 (16.92, 19.40)	15.70 (14.42, 17.06)	
Patient comorbidities					
Family history of CAD	17.55 (17.05, 18.06)	19.12 (18.43, 19.83)	18.06 (17.49, 18.64)	12.88 (12.13, 13.66)	<0.001
Prior MI	17.45 (16.75, 18.17)	16.14 (15.56, 16.74)	18.19 (17.19, 19.23)	18.11 (17.33, 18.92)	<0.001
Prior PCI	19.37 (18.35, 20.44)	18.31 (17.48, 19.17)	20.26 (19.02, 21.56)	19.13 (17.83, 20.52)	<0.001
Prior CABG	13.97 (13.40, 14.57)	11.99 (11.60, 12.39)	14.31 (13.40, 15.27)	17.17 (16.25, 18.12)	<0.001
Prior stroke or TIA	7.64 (7.20, 8.10)	6.60 (6.24, 6.98)	7.90 (7.37, 8.46)	9.08 (8.16, 10.09)	<0.001
Smoking	52.52 (51.39, 53.63)	53.71 (52.86, 54.55)	53.27 (52.03, 54.51)	47.95 (45.71, 50.20)	<0.001
Dyslipidemia	74.11 (73.69, 74.51)	73.41 (72.71, 74.10)	75.35 (74.84, 75.86)	72.11 (71.23, 72.98)	<0.001
Obesity	21.85 (20.94, 22.80)	20.98 (20.31, 21.67)	22.37 (21.09, 23.70)	22.25 (21.15, 23.38)	0.004
Diabetes mellitus	42.30 (41.85, 42.74)	37.76 (37.05, 38.47)	41.82 (41.04, 42.60)	53.07 (52.20, 53.95)	<0.001
Hypertension	58.77 (58.28, 59.26)	62.37 (61.63, 63.10)	60.75 (59.89, 61.60)	45.81 (44.64, 46.99)	<0.001
Carotid artery disease	2.08 (1.97, 2.20)	1.46 (1.33, 1.61)	1.94 (1.72, 2.18)	3.78 (3.49, 4.10)	<0.001
Peripheral vascular disease	11.47 (10.89, 12.08)	9.25 (8.78, 9.76)	11.30 (10.75, 11.87)	16.59 (14.95, 18.37)	<0.001
Congestive heart failure	23.58 (23.09, 24.07)	19.29 (18.78, 19.81)	21.20 (20.42, 22.00)	39.07 (38.03, 40.13)	<0.001
Valvular disease	11.92 (11.51, 12.35)	9.09 (8.51, 9.72)	11.15 (10.70, 11.62)	19.93 (18.89, 21.01)	<0.001
Chronic pulmonary disease	20.49 (20.05, 20.93)	17.23 (16.73, 17.75)	20.03 (19.55, 20.51)	28.53 (27.43, 29.67)	<0.001
Chronic renal disease	22.01 (21.69, 22.34)	15.94 (15.49, 16.40)	20.81 (20.15, 21.48)	37.98 (37.08, 38.88)	<0.001
Atrial fibrillation	14.28 (13.89, 14.67)	10.71 (10.35, 11.08)	14.27 (13.76, 14.79)	21.72 (20.27, 23.23)	<0.001
Cardiogenic shock	2.81 (2.70, 2.92)	3.15 (2.83, 3.52)	1.92 (1.70, 2.17)	4.53 (4.18, 4.91)	<0.001
Use of IABP	1.78 (1.67, 1.90)	1.97 (1.64, 2.38)	1.22 (1.13, 1.32)	2.94 (2.55, 3.39)	<0.001
Procedure characteristic					
Number of treated lesions					<0.001
1	80.82 (80.44, 81.19)	82.72 (82.17, 83.26)	81.19 (80.65, 81.72)	75.81 (74.95, 76.66)	
2	16.14 (15.83, 16.47)	14.91 (14.46, 15.36)	16.09 (15.63, 16.56)	18.87 (18.15, 19.61)	
3	2.53 (2.39, 2.68)	2.03 (1.86, 2.21)	2.27 (2.04, 2.54)	4.29 (3.94, 4.67)	
≥4	0.51 (0.46, 0.56)	0.34 (0.29, 0.41)	0.44 (0.38, 0.52)	1.02 (0.80, 1.31)	
Hospital characteristics					
Hospital location					0.001
Rural	5.85 (5.29, 6.46)	6.04 (5.54, 6.59)	5.64 (5.01, 6.35)	6.02 (5.11, 7.07)	
Urban nonteaching	20.02 (18.34, 21.81)	20.94 (19.55, 22.41)	20.07 (18.14, 22.16)	17.93 (15.79, 20.30)	
Urban teaching	74.13 (71.87, 76.27)	73.01 (71.22, 74.73)	74.28 (71.66, 76.74)	76.05 (73.16, 78.72)	
Hospital bed size					<0.001
Small	16.45 (14.35, 18.80)	16.95 (14.89, 19.22)	16.02 (13.72, 18.63)	16.62 (14.24, 19.31)	
Medium	29.69 (27.13, 32.39)	30.99 (28.94, 33.11)	29.78 (26.82, 32.93)	26.75 (23.82, 29.90)	
Large	53.86 (49.97, 57.70)	52.07 (49.12, 55.01)	54.20 (49.70, 58.63)	56.63 (52.03, 61.12)	
Region					0.001
Northeast	22.61 (16.94, 29.50)	22.10 (17.90, 26.95)	22.79 (16.30, 30.90)	23.19 (16.59, 31.43)	
Midwest	23.93 (21.94, 26.04)	24.45 (22.87, 26.10)	24.69 (22.31, 27.23)	20.76 (18.56, 23.13)	
South	42.32 (38.84, 45.86)	40.65 (38.16, 43.18)	41.24 (37.31, 45.28)	48.76 (43.79, 53.75)	
West	11.14 (10.17, 12.20)	12.81 (11.85, 13.83)	11.28 (10.15, 12.52)	7.30 (6.45, 8.24)	

Abbreviations: CABG, Coronary artery bypass grafting; CAD, Coronary artery disease; IABP, Intra‐aortic balloon pump; MI, Myocardial infarction; NSTEMI, Non‐ST elevation myocardial infarction; PCI, Percutaneous coronary intervention; SD, Standard deviation; TIA, Transient ischemic attack.

### 3.2 Study outcomes

The incidence of NAIS was twice as high in individuals who had late PCI compared with those who had early and medium PCI (1.30% vs. 0.63% vs. 0.71%; *p* < 0.001) (Table 2). Patients who underwent late PCI were associated with a longer length of hospital stay than those who underwent early and medium PCI, with a median of 7.00 versus 2.38 and 3.30 days, respectively (*p* < 0.001), and higher total hospital charges, with a median of $137,143.05 versus $90,202.71 and $94,160.05, respectively (*p* < 0.001) (Table 2). After controlling for confounders, multivariate regression revealed that compared with the patients with early PCI, only late PCI was significantly associated with a 54% increased NAIS risk (adjusted odds ratio [aOR]: 1.54; 95% confidence interval [CI]: 1.29–1.84, *p* < 0.001) (Table 3).

**Table 2:** In‐hospital outcomes in patients with NSTEMI undergoing PCI among the early PCI, medium PCI, and late PCI groups.

	Total (%) 95% CI	Early PCI (%) 95% CI	Medium PCI (%) 95% CI	Late PCI (%) 95% CI	*p*‐value
n	633,115	225,570	299,235	108,310	
New‐onset acute ischemic stroke	0.78 (0.72, 0.85)	0.63 (0.51, 0.76)	0.71 (0.63, 0.80)	1.30 (1.11, 1.51)	<0.001
Length of stay (days)	3.61 (3.57, 3.64)	2.38 (2.35, 2.42)	3.30 (3.25, 3.35)	7.00 (6.86, 7.14)	<0.001
Total hospital cost ($)	100,097.61 (96,152.95, 104,042.27)	90,202.71 (87,590.65, 92,814.77)	94,160.05 (89,583.00, 98,737.10)	137,143.05 (130,465.14, 143,820.96)	<0.001

Abbreviations: NSTEMI, non‐ST elevation myocardial infarction; PCI, percutaneous coronary intervention.

**Table 3:** NAIS risk in patients with non‐ST elevation myocardial infarction (NSTEMI) undergoing PCI among the early PCI, medium PCI, and late PCI groups.

	Non‐adjusted model			Adjusted model		
	OR	95% CI	P‐value	OR	95% CI	P‐value
Early PCI	1.0			1.0		
Medium PCI	1.13	0.97, 1.31	0.111	1.10	0.94, 1.28	0.242
Late PCI	2.08	1.76, 2.46	<0.001	1.54	1.29, 1.84	<0.001

*Note*: Variables included in the adjusted model included age, sex, race, weekday admission, primary expected payer, household income (median), hospital location, hospital bed size, region, prior MI, prior PCI, prior CABG, prior stroke or TIA, smoking, dyslipidemia, obesity, diabetes mellitus, hypertension, carotid artery disease, peripheral vascular disease, congestive heart failure, valvular disease, chronic pulmonary disease, chronic renal disease, atrial fibrillation, cardiogenic shock, number of treated lesions, and use of IABP.

Abbreviations: CI, Confidence interval; NAIS, new‐onset acute ischemic stroke; OR, Odds ratio; PCI, percutaneous coronary intervention.

### 3.3 Subgroup analysis

To further confirm the robustness of our findings (Table 3), we performed stratified analyses by subgroups defined by major covariables that affected stroke risk in our study. The results presented in Table 4 are highly consistent regardless of the subgroup; late PCI increased the risk of NAIS compared with early PCI in patients with NSTEMI. Late PCI was associated with a 1.17‐fold (aOR:2.17, 95% CI:1.53–3.06) increased risk of NAIS compared with early PCI in patients aged <65 years and a 37% (aOR:1.37, 95% CI:1.11–1.68) increased risk of NAIS in older patients (≥65 years). Similarly, men were significantly more likely to develop NAIS (aOR: 1.73, 95% CI: 1.35–2.21) than women (aOR: 1.31, 95% CI: 1.02–1.70). Significant interactions were observed in age (*p* for interaction <0.001) and sex (interaction‐value *p* = 0.040).

**Table 4:** Subgroup analysis of the NAIS risk in patients with non‐ST elevation myocardial infarction (NSTEMI) undergoing PCI among the early PCI, medium PCI, and late PCI groups.

	n	Early PCI	Medium PCI			Late PCI			*p*‐value for interaction
			OR	95% CI	*p*‐value	OR	95% CI	*p*‐value	
		1	2			3			
Age (y)									<0.001
<65	57,608	1.0	1.13	0.83, 1.54	0.428	2.17	1.53, 3.06	<0.001	
≥65	69,058	1.0	1.07	0.90, 1.29	0.429	1.37	1.11, 1.68	0.003	
Sex									0.040
Male	82,753	1.0	1.13	0.91, 1.41	0.264	1.73	1.35, 2.21	<0.001	
Female	43,913	1.0	1.06	0.85, 1.33	0.584	1.31	1.02, 1.70	0.036	
Past stroke or TIA									0.900
No	116,992	1.0	1.09	0.93, 1.28	0.297	1.50	1.24, 1.81	<0.001	
Yes	9674	1.0	1.21	0.74, 1.97	0.446	1.87	1.09, 3.20	0.022	
Diabetes mellitus									0.474
No	73,090	1.0	1.08	0.88, 1.33	0.483	1.64	1.29, 2.10	<0.001	
Yes	53,576	1.0	1.09	0.86, 1.38	0.473	1.44	1.11, 1.87	0.006	
Chronic renal disease									0.082
No	98,791	1.0	1.10	0.92, 1.31	0.302	1.65	1.34, 2.04	<0.001	
Yes	27,875	1.0	1.03	0.76, 1.41	0.833	1.25	0.90, 1.73	0.179	
Atrial fibrillation									0.010
No	108,587	1.0	1.14	0.95, 1.36	0.160	1.74	1.42, 2.14	<0.001	
Yes	18,079	1.0	0.96	0.71, 1.31	0.806	1.08	0.76, 1.52	0.672	
Valvular disease									0.083
No	111,568	1.0	1.05	0.89, 1.24	0.550	1.55	1.28, 1.88	<0.001	
Yes	15,098	1.0	1.49	0.95, 2.34	0.086	1.68	1.04, 2.73	0.035	
Use of IABP									0.045
No	124,409	1.0	1.10	0.94, 1.30	0.228	1.60	1.33, 1.92	<0.001	
Yes	2257	1.0	1.27	0.69, 2.34	0.445	0.94	0.47, 1.87	0.864	

Abbreviations: CI, Confidence interval; IABP, Intra‐aortic balloon pump; NAIS, New‐onset acute ischemic stroke; NSTEMI, Non‐ST elevation myocardial infarction; OR, Odds ratio; PCI, Percutaneous coronary intervention; TIA, Transient ischemic attack. Covariates were adjusted as in the adjusted model (Table 3).

### 3.4 Temporal trends in the incidence of NAIS

**Figure 2:**
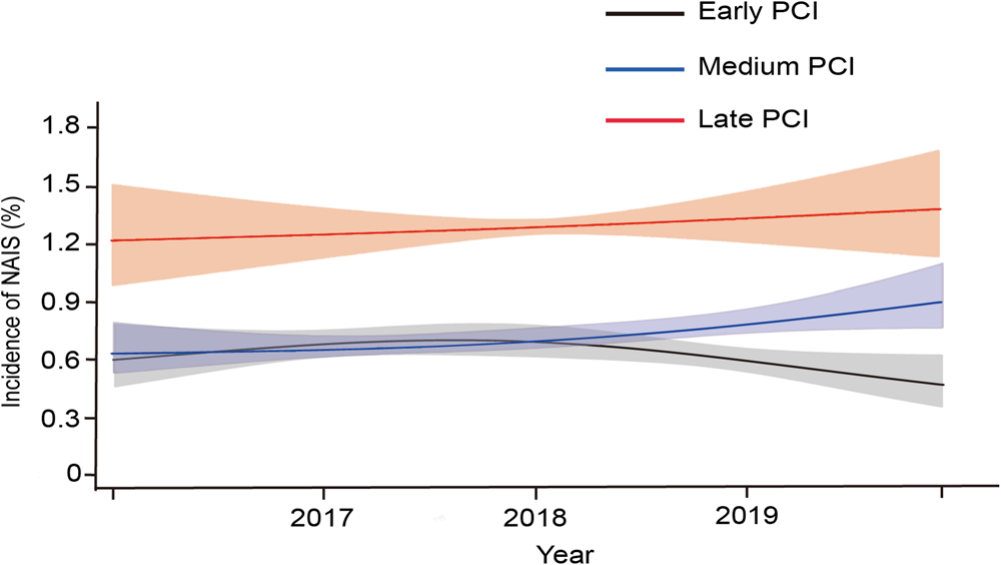
Temporal trends in the incidence of post‐PCI NAIS in non‐ST elevation myocardial infarction (NSTEMI) patients undergoing PCI among early PCI, medium PCI, and late PCI groups. The yearly NAIS rates for patients with NSTEMI who had undergone PCI surgery increased in both the medium (ranging from 0.62% to 0.89%) and late PCI (ranging from 1.23% to 1.39%) groups, but declined in the early PCI group (ranging from 0.60% to 0.47%). Shaded areas are 95% CI. CI, Confidence interval; NAIS, New‐onset acute ischemic stroke; NSTEMI, Non‐ST elevation myocardial infarction; PCI, Percutaneous coronary intervention.

From 2016 to 2019, the yearly NAIS rates for patients with NSTEMI who had undergone PCI surgery increased in both the medium (ranging from 0.62% to 0.89%) and late PCI (ranging from 1.23% to 1.39%) groups but declined in the early PCI group (ranging from 0.60% to 0.47%). Throughout the research period, the NAIS rates for patients in the late PCI group (*p*_trend_ = 0.152) were consistently higher than those for the patients in the early (*p*_trend_ = 0.048) and medium groups (*p*_trend_ = 0.579) (Figure 2).

## 4 DISCUSSION

In a nationally representative database, we assessed the risk of NAIS in patients hospitalized with NSTEMI undergoing PCI among three groups (early PCI, medium PCI, and late PCI). Our main findings are as follows: (1) Late PCI was associated with a higher risk of NAIS, more costly hospitalizations, and longer hospital stays; (2) late PCI significantly increased the risk of NAIS in men and patients aged <65 years; and (3) the incidence of NAIS in the late PCI group has increased significantly over the past 4 years.

Our research showed that late PCI increased the likelihood of NAIS compared with early PCI. However, a previous RCT (TIMACS) study^13^ and the latest meta‐analysis^12^ showed that a delayed invasive strategy did not increase the risk of NAIS compared with an early invasive strategy in patients with acute coronary syndrome. The invasive strategy included PCI and CABG in previous studies, and patients who chose CABG due to the lesion complexity carried a higher risk of postprocedural NAIS.^16^, ^17^ This study is unique in that it exclusively concerns patients with NSTEMI treated with PCI only to eliminate the effects of CABG in the contemporary era. The median time of the delayed invasive strategy was within 72 h in the previous analysis. Our study showed that patients undergoing late PCI accounted for a considerably higher proportion of the total patients included in the study (17.10%); therefore, this group of patients should not be ignored. We noted that patients who underwent late PCI had a much higher risk profile (older, more female, more comorbidities of previous stroke history, diabetes mellitus, renal disease, atrial fibrillation, valvular disease, and more circulatory support devices) than those who underwent early PCI in our study, and all of the risk factors are independent predictive risk factors for postoperative NAIS.^4^, ^5^, ^18^, ^19^, ^20^, ^21^, ^22^ Meanwhile, previous studies demonstrated that patients with high‐risk characteristics were more likely to suffer from NAIS, which may explain the higher risk of NAIS in patients with late PCI.^5^ Plateletcrit, a marker of cardiovascular disease, has recently become an interesting topic in cardiovascular research. One study discovered that higher plateletcrit was associated with increased long‐term mortality and adverse stroke outcomes in patients with carotid stenosis.^23^ In our study, the late PCI group had the highest proportion of patients with carotid artery disease. Patients in the late PCI group had a higher plateletcrit due to the use of antiplatelet drugs and longer anticoagulation times, which may lead to a higher risk of adverse outcomes.^24^ Our study also showed that late PCI had the longest hospitalization and greatest hospitalization costs, which are consistent with the results of previous studies.^12^, ^25^ Hence, given the high clinical risk profile and heavy burden of patients with late PCI, further investigations are needed to improve outcomes.

We also found a higher NAIS risk in patients aged <65 years than in patients aged ≥65 years. Although stroke was previously thought to be a disease of the elderly, a growing trend of strokes in young individuals has recently been observed.^26^, ^27^, ^28^ Our results also revealed a relatively high prevalence of unhealthy lifestyle behaviors (e.g., smoking, drug abuse, obesity, and hypertension) among young patients (Table S2), which was also observed in a previous study.^28^ Moreover, the presence of multiple risk factors shifted toward a greater NAIS risk in young adults. Emphasizing early identification and proactive preventative methods is critical because of the significant personal, societal, and economic repercussions for young people.^29^ We also found an increased risk of NAIS in men than in women. Indeed, previous studies have found that the coronary artery of men is prone to harbor complicated lesions.^30^, ^31^ Previous studies have also suggested that patients with myocardial infarction with prior CABG have a higher risk profile than those without prior CABG.^32^ Our study showed that men had higher procedural complications (prior PCI and prior CABG) than women (Table S3). When acute myocardial infarction reoccurs, the vascular lesions are more severe. Patients who received PCI for complicated coronary artery lesions had poorer clinical outcomes than those without complicated coronary artery lesions.^33^, ^34^ In addition, estrogen has various protective benefits in female patients, including protecting myocardium cells, lowering myocardial cell death, and preventing plaque rupture.^35^ Furthermore, men acquire atherosclerotic plaques sooner, and the plaques are more inflammatory and contain extra unstable characteristics, leading to an increase in stroke occurrences in men.^36^ Patients under the age of 65 and men warrant more attention, including active treatment of associated complications and the improvement of poor lifestyle habits, which could reduce their risk of developing NAIS.

Our study found an increased temporal trend of NAIS incidence from 2016 to 2019. In previous studies, the incidence of post‐PCI NAIS increased significantly in patients who had undergone PCI previously.^3^, ^22^ Our study showed the same temporal trend and showed that patients with late PCI had a consistently higher incidence of NAIS than those with early and medium PCI during the study period, which can be explained by the higher clinical risk of patients treated with late PCI. Nonetheless, further research is required to validate and examine the underlying reasons for these concerning trends.

### 4.1 Strengths

Our research has various advantages. The NIS data set consists of a 20% stratified sample of all community hospital discharges in the United States, representing a nationwide, real‐world experience, which includes high‐risk patients who are frequently excluded from RCTs. Even though the NIS is an administrative database, the enormous sample size of weighted patients, which confers appropriate power to catch relatively infrequent PCI complications, makes this database a good source to investigate our outcome of interest. In the present study, we considered age, sex, comorbidities of previous stroke history, diabetes mellitus, renal disease, atrial fibrillation, valvular heart disease, and circulatory support devices as confounders. Subgroup analysis revealed that the increased risk of NAIS in the late PCI group was stable and that this risk was increased in men and those aged <65 years.

### 4.2 Limitations

This study also has various limitations that warrant discussion. First, because this is a retrospective, observational study, the risk of selection bias and residual estimable and inestimable confounding cannot be ruled out. Second, because the NIS is an administrative database, there is the possibility of unnoticed errors in assigning diagnostic and procedural codes. Third, NIS cannot determine the temporal sequence of diagnostic and procedural codes. There likely have been patients who were included in the late group because of the NAIS itself and the treating physician decided to delay the procedure until recovery. Fourth, the unavailability of PCI success rate data within the NIS database precluded an assessment of potential variations in procedural success among the three groups. Fifth, owing to the absence of prehospital data within the NIS database, including symptom‐to‐balloon times, we were unable to assess the potential impact of prehospital delays on outcomes for the three groups. Sixth, data on the specific location of the infarct‐related artery (left main, left anterior descending, left circumflex, or right coronary artery) and the location of the treated vessel were not available in the NIS database, representing a potential avenue for further investigation. Seventh, the NIS does not contain laboratory results (e.g., coagulation function), imaging results (e.g., resonance imaging and CT scan), and use of drugs (e.g., anticoagulant and antiplatelet medications). Eighth, because the database does not provide the time of the procedure in hours, we used the “PRDAY” variable to define the three study groups. Finally, the outcomes in the NIS database are limited to in‐hospital events and cannot be used to compare different timings of PCI for long‐term outcomes.

## 5 CONCLUSIONS

Late PCI was associated with a higher risk of NAIS than early PCI in patients with NSTEMI, particularly in men and those aged <65 years. Further studies are needed to identify effective prevention and management strategies given the high risk of NAIS in patients with late PCI.

## AUTHOR CONTRIBUTIONS

**Bo Shi**: Formal analysis; Writing—original draft. **Xueping Ma**: Funding acquisition; Resources; Supervision. **Congyan Ye**: Formal analysis; Software. **Rui Yan**: Formal analysis; Software. **Shizhe Fu**: Formal analysis; Software. **Kairu Wang**: Formal analysis; Software. **Mingzhi Cui**: Investigation; Project administration. **Ru Yan**: Funding acquisition; Resources; Supervision. **Shaobin Jia**: Funding acquisition; Resources; Supervision. **Guangzhi Cong**: Formal analysis; Writing—original draft.

## CONFLICT OF INTEREST STATEMENT

The authors report no affiliation or involvement in potential conflicts of interest. The funding source had no involvement in study design, collection, analysis, interpretation of the data, writing of the report, nor the decision to submit the report for publication.

## ETHICS STATEMENT

Because of the public nature of the NIS database (www.hcup-us.ahrq.gov) and the lack of any individually identifiable information, the Institutional Review Board waived the requirement for informed consent under the Health Insurance Portability and Accountability Act. All authors have read and approved the final version of the manuscript. Guangzhi Cong and Bo Shi had full access to all of the data in this study and takes complete responsibility for the integrity of the data and the accuracy of the data analysis.

## TRANSPARENCY STATEMENT

The lead author Bo Shi, Shizhe Fu, Guangzhi Cong affirms that this manuscript is an honest, accurate, and transparent account of the study being reported; that no important aspects of the study have been omitted; and that any discrepancies from the study as planned (and, if relevant, registered) have been explained.

## Supporting information

Supporting information.

## Data Availability

NIS database available online (www.hcup-us.ahrq.gov). The data that support the findings of this study are openly available in The National (Nationwide) Inpatient Sample (NIS) at https://hcup-us.ahrq.gov/nisoverview.jsp.
