# Supplementary material for: Timing of percutaneous coronary intervention and risk of new‐onset acute ischemic stroke in non‐ST elevation myocardial infarction: A retrospective cohort study insight into the National Inpatient Sample Database (2016–2019)
Source: Health Sci Rep. 2024 Sep 18;7(9):e70029. doi: 10.1002/hsr2.70029 (PMC11409050; doi:10.1002/hsr2.70029)
Supplement: Supplementary file 1 — Supporting information. [file HSR2-7-e70029-s001.docx]

**Supplemental Material**

Table S1. International classification of diseases and clinical classification software codes for the identification of clinical variables

|  | ICD-10-CM code |
| --- | --- |
| Family history of CAD | Z8249 |
| Prior MI | I252 and I22XX |
| Prior PCI | Z9861 and Z955 |
| Prior CABG | Z951, I257XX, I25810, and I25812 |
| Prior stroke or TIA | Z8673 |
| Smoking | F17X, Z720, and Z87891 |
| Dyslipidemia | E78X |
| Obesity | E66X |
| Diabetes mellitus | E10X, E11X, E12X, E13X, and E14X |
| Hypertension | I10X, I11X, I12X, I13X, and I15X |
| Carotid artery disease | I652X |
| Peripheral vascular disease | I70X, I71X, I731, I738, I739, I771, I790, I792, K551, K558, K559, Z958, and Z959 |
| Congestive heart failure | I50X, I110, I130, I132, I255, I43X, I420, I425, I426, I427, I428, I429, P290, and I099 |
| Valvular disease | A520, I091, I098, Q230, Q231, Q232, Q233, Z952, Z954, I05X, I06X, I07X, I08X, I34X, I35X, I36X, I37X, I38X, and I39X |
| Chronic pulmonary disease | I278, I279, J684, J701, J703, J40X, J41X, J42X, J43X, J44X, J45X, J46X, J47X, J60X, J61X, J62X, J63X, J64X, J65X, J66X, and J67X |
| Chronic renal disease | I120, I131, N250, Z490, Z491, Z492, Z940, Z992, N18X, and N19X |
| Atrial fibrillation | I480, I481, I4811, I4819, I4820, I4821, I489, and I4891 |
| Cardiogenic shock | R570 |
| Use of IABP | 5A02110 and 5A02210 |
| Drug abuse | F11X, F12X, F13X, F14X, F15X, F16X, F18X, F19X, and Z7151 |

Abbreviations: PCI: Percutaneous coronary intervention, CAD: Coronary artery disease, MI: Myocardial infarction, TIA: Transient ischemic attack, CABG: Coronary artery bypass grafting, and IABP: Intra-aortic balloon pump.

Table S2. Baseline demographics, comorbidities, and hospital characteristics of patients with NSTEMI undergoing PCI between age ≤65-year and age >65-year groups

|  | Age ≤ 65 (%) | Age > 65 (%) | P-value |
| --- | --- | --- | --- |
| n | 57,608 | 69,058 |  |
| Patient demographics |  |  |  |
| Age, mean, y (SD) | 54.53 ± 7.32 | 75.09 ± 7.02 | <0.001 |
| Female | 16,822 (29.20) | 27091 (39.23) | <0.001 |
| Race |  |  | <0.001 |
| White | 41,590 (72.19) | 56,120 (81.27) |  |
| Black | 7697 (13.36) | 5268 (7.63) |  |
| Hispanic | 5037 (8.74) | 4421 (6.40) |  |
| Other | 3284 (5.70) | 3249 (4.70) |  |
| Primary expected payer |  |  | <0.001 |
| Medicare/Medicaid | 19,483 (33.82) | 60,816 (88.07) |  |
| Private insurance | 29,478 (51.17) | 6017 (8.71) |  |
| Self-pay | 5447 (9.46) | 583 (0.84) |  |
| No charge/other | 3200 (5.55) | 1642 (2.38) |  |
| Weekend admission | 14,796 (25.68) | 17,094 (24.75) | <0.001 |
| Household income (median) |  |  | <0.001 |
| 0–25th percentile | 18,727 (32.51) | 19,475 (28.20) |  |
| 26–50th percentile | 15,780 (27.39) | 19,166 (27.75) |  |
| 51–75th percentile | 13,298 (23.08) | 17,245 (24.97) |  |
| 76–100th percentile | 9803 (17.02) | 13,172 (19.07) |  |
| Patient comorbidities |  |  |  |
| Family history of CAD | 12,901 (22.39) | 9325 (13.50) | <0.001 |
| Prior MI | 9330 (16.20) | 12,765 (18.48) | <0.001 |
| Prior PCI | 9811 (17.03) | 14,726 (21.32) | <0.001 |
| Prior CABG | 4885 (8.48) | 12,811 (18.55) | <0.001 |
| Prior stroke or TIA | 2959 (5.14) | 6715 (9.72) | <0.001 |
| Smoking | 35,133 (60.99) | 31,392 (45.46) | <0.001 |
| Dyslipidemia | 41,207 (71.53) | 52,656 (76.25) | <0.001 |
| Obesity | 15,998 (27.77) | 11,686 (16.92) | <0.001 |
| Diabetes mellitus | 22,517 (39.09) | 31,059 (44.98) | <0.001 |
| Hypertension | 34,755 (60.33) | 39,689 (57.47) | <0.001 |
| Carotid artery disease | 544 (0.94) | 2094 (3.03) | <0.001 |
| Peripheral vascular disease | 4343 (7.54) | 10,188 (14.75) | <0.001 |
| Congestive heart failure | 10,368 (18.00) | 19,494 (28.23) | <0.001 |
| Valvular disease | 3570 (6.20) | 11,528 (16.69) | <0.001 |
| Chronic pulmonary disease | 9974 (17.31) | 15,977 (23.14) | <0.001 |
| Chronic renal disease | 7483 (12.99) | 20,392 (29.53) | <0.001 |
| Atrial fibrillation | 3363 (5.84) | 14,716 (21.31) | <0.001 |
| Cardiogenic shock | 1110 (1.93) | 2443 (3.54) | <0.001 |
| Use of IABP | 752 (1.31) | 1505 (2.18) | <0.001 |
| Drug abuse | 3382 (5.87) | 669 (0.97) | <0.001 |
| Hospital characteristics |  |  |  |
| Hospital location |  |  | 0.020 |
| Rural | 3439 (5.97) | 3977 (5.76) |  |
| Urban non-teaching | 11,690 (20.29) | 13,687 (19.82) |  |
| Urban teaching | 42,479 (73.74) | 51,394 (74.42) |  |
| Hospital bed size |  |  | <0.001 |
| Small | 9668 (16.78) | 11,192 (16.21) |  |
| Medium | 16,822 (29.20) | 20,782 (30.09) |  |
| Large | 31,118 (54.02) | 37,084 (53.70) |  |
| Region |  |  | <0.001 |
| Northeast | 12,334 (21.41) | 16,317 (23.63) |  |
| Midwest | 13,815 (23.98) | 16,493 (23.88) |  |
| South | 25,550 (44.35) | 28,046 (40.61) |  |
| West | 5909 (10.26) | 8202 (11.88) |  |

Values are mean ± SD or n (%).

Abbreviations: SD: Standard deviation, PCI: Percutaneous coronary intervention, CAD: Coronary artery disease, MI: Myocardial infarction, TIA: Transient ischemic attack, CABG: Coronary artery bypass grafting, IABP: Intra-aortic balloon pump, and NSTEMI: Non-ST elevation myocardial infarction.

Table S3. Baseline demographics, comorbidities, and hospital characteristics of patients with NSTEMI undergoing PCI between women and men

|  | Male (%) | Female (%) | P-value |
| --- | --- | --- | --- |
| n | 82,753 | 43,913 |  |
| Patient demographics |  |  |  |
| Age, mean, y (SD) | 64.66 ± 12.26 | 67.76 ± 12.67 | <0.001 |
| Race |  |  | <0.001 |
| White | 64,912 (78.44) | 32,798 (74.69) |  |
| Black | 7136 (8.62) | 5829 (13.27) |  |
| Hispanic | 6250 (7.55) | 3208 (7.31) |  |
| Other | 4455 (5.38) | 2078 (4.73) |  |
| Primary expected payer |  |  | <0.001 |
| Medicare/Medicaid | 48,186 (58.23) | 32,113 (73.13) |  |
| Private insurance | 26,409 (31.91) | 9086 (20.69) |  |
| Self-pay | 4284 (5.18) | 1746 (3.98) |  |
| No charge/other | 3874 (4.68) | 968 (2.20) |  |
| Weekend admission | 20,955 (25.32) | 10,935 (24.90) | 0.101 |
| Household income (median) |  |  | <0.001 |
| 0–25th percentile | 23,662 (28.59) | 14,540 (33.11) |  |
| 26–50th percentile | 22,549 (27.25) | 12,397 (28.23) |  |
| 51–75th percentile | 20,413 (24.67) | 10,130 (23.07) |  |
| 76–100th percentile | 16,129 (19.49) | 6846 (15.59) |  |
| Patient comorbidities |  |  |  |
| Family history of CAD | 14,528 (17.56) | 7698 (17.53) | 0.909 |
| Prior MI | 15,223 (18.40) | 6872 (15.65) | <0.001 |
| Prior PCI | 16,948 (20.48) | 7589 (17.28) | <0.001 |
| Prior CABG | 13,202 (15.95) | 4494 (10.23) | <0.001 |
| Prior stroke or TIA | 5561 (6.72) | 4113 (9.37) | <0.001 |
| Smoking | 46,199 (55.83) | 20,326 (46.29) | <0.001 |
| Dyslipidemia | 61,936 (74.84) | 31,927 (72.71) | <0.001 |
| Obesity | 17,123 (20.69) | 10,561 (24.05) | <0.001 |
| Diabetes mellitus | 32,874 (39.73) | 20,702 (47.14) | <0.001 |
| Hypertension | 49,356 (59.64) | 25,088 (57.13) | <0.001 |
| Carotid artery disease | 1557 (1.88) | 1081 (2.46) | <0.001 |
| Peripheral vascular disease | 73,279 (88.55) | 38,856 (88.48) | 0.720 |
| Congestive heart failure | 19,384 (23.42) | 10,478 (23.86) | 0.081 |
| Valvular disease | 8690 (10.50) | 6408 (14.59) | <0.001 |
| Chronic pulmonary disease | 14,942 (18.06) | 11,009 (25.07) | <0.001 |
| Chronic renal disease | 17,743 (21.44) | 10,132 (23.07) | <0.001 |
| Atrial fibrillation | 11,795 (14.25) | 6284 (14.31) | 0.783 |
| Cardiogenic shock | 2160 (2.61) | 1393 (3.17) | <0.001 |
| Use of IABP | 1418 (1.71) | 839 (1.91) | 0.012 |
| Drug abuse | 3100 (3.75) | 951 (2.17) | <0.001 |
| Hospital characteristics |  |  |  |
| Hospital location |  |  | 0.021 |
| Rural | 4735 (5.72) | 2681 (6.11) |  |
| Urban non-teaching | 16,585 (20.04) | 8792 (20.02) |  |
| Urban teaching | 61,433 (74.24) | 32,440 (73.87) |  |
| Hospital bed size |  |  | 0.933 |
| Small | 13,618 (16.46) | 7242 (16.49) |  |
| Medium | 24,546 (29.66) | 13,058 (29.74) |  |
| Large | 44,589 (53.88) | 23,613 (53.77) |  |
| Region |  |  | <0.001 |
| Northeast | 19,150 (23.14) | 9501 (21.64) |  |
| Midwest | 19,548 (23.62) | 10,760 (24.50) |  |
| South | 34,464 (41.65) | 19,132 (43.57) |  |
| West | 9591 (11.59) | 4520 (10.29) |  |

Values are mean ± SD or N (%).

Abbreviations: SD: Standard deviation, PCI: Percutaneous coronary intervention, CAD: Coronary artery disease, MI: Myocardial infarction, TIA: Transient ischemic attack, CABG: Coronary artery bypass grafting, IABP: Intra-aortic balloon pump, and NSTEMI: Non-ST elevation myocardial infarction.
